# Monostotic fibrous dysplasia involving occipital bone: a case report and review of literature

**DOI:** 10.11604/pamj.2014.19.124.5203

**Published:** 2014-10-03

**Authors:** Recep Basaran, Mustafa Kaksi, Erdal Gur, Mustafa Efendioglu, Ece Balkuv, Aydin Sav

**Affiliations:** 1 Dr. Lutfi Kirdar Kartal Education and Research Hospital, Department of Neurosurgery, Istanbul, Turkey; 2 Eyup State Hospital, Department of Neurosurgery, Istanbul, Turkey; 3 Istanbul Medeniyet University Goztepe Education and Research Hospital, Department of Neurology, Istanbul, Turkey; 4 Acibadem University School of Medicine, Department of Pathology, Istanbul, Turkey

**Keywords:** Fibrous dysplasia, cystic, cranial, monostotic, occipital, trauma

## Abstract

Fibrous dysplasia (FD) is a progressive systemic bone tumour of young and it can be seen on cranial bones. FD is divided into three types according to radiological features. The second most common subtype is polyostotic subtype. With this article, we aimed to review and present clinical features, radiological examination, differential diagnosis and treatment management of a case of solitary monostotic fibrous dysplasia of occipital bone. 15 years old female patient admitted to our hospital for a bump and in the back of his head that she noticed 1 month ago. Her physical and neurological examination was normal. On cranial CT examination we detected a bony defect. Her gadolinium enhanced cranial MRI revealed bony defect along with massive gadolinium enhancement in adjacent tissue. On histopathologic examination; PANCK, CD68, CD1a were found negative and CD45, S-100, Vimentine were found positive. Ki-67 was 4,8%. In conclusion, fibrous dysplasia is a progressive bone disease of the young patients. Despite its resemblance to a benign lesion by not being symptomatic it can progress and cause severe bony defects and skin lesions. Total surgical resection is necessary and sufficient for total treatment.

## Introduction

Fibrous dysplasia (FD) is a systemic bone tumor of young patients involving cranial bones [1]. Of all cranial bones, mostly it involves facial or frontal bones and basis cranium and rarely it can be seen on occipital bone and convexities [2]. Even though the pathogenesis of fibrous dysplasia is not known, lately some authors found genetical abnormalities responsible in the pathogenesis [3]. Radiologically, Fries defines fibrous dysplasia of cranium in 3 subtypes [4]. The rarest subtype with a frequency of 21% obtains a dens bone tissue surrounding areas of cystic differentiations. This type of fibrous dysplasia is formed by many different fibrous elements [4]. Today, there are three defined subtypes of fibrous dysplasia: monostotic, polyostotic and McCune Albright syndrome. Most common subtype is monostotic subtype with a frequency of 70% and it is also the most benign form. Monostotic subtype is common between ages 20-30 and involves one bone, usually costal or craniofacial bones. It is usually asymptomatic and if becomes symptomatic usually it is revealed with skin lesion secondary to mass effect of the lesion [5]. For radiological evaluation of fibrous dysplasia, first modality is CT imaging. CT reveals bony elements in detail. As for the evaluation of adjacent tissue, soft tissue and fibrous components, especially on cystic subtype, MRI is superior to CT. With this article, we aimed to review and present clinical features, radiological examination, differential diagnosis and treatment management of a case of solitary monostotic fibrous dysplasia of occipital bone.

## Patient and observation

**Figure 1:**
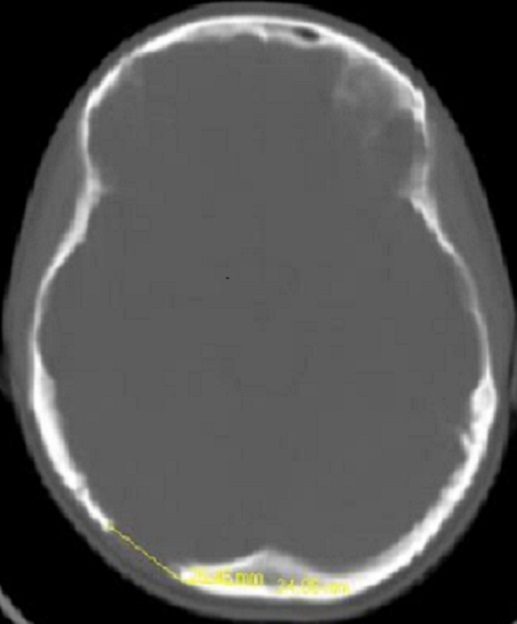
Cranial bone CT image of 3x3 cm bone defect on right occipital bone

**Figure 2:**
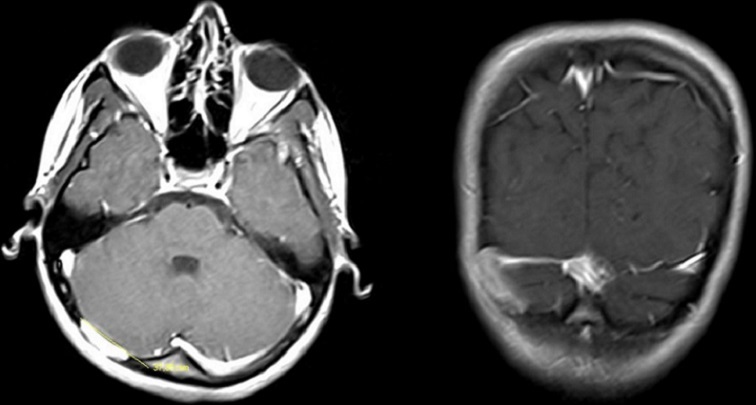
Gadolium enhancing cranial MR image of bone defect and surrounding lesion with high gadolinium enhancement

15 years old female patient admitted to our hospital for a bump and in the back of his head that she noticed 1 month ago. She has a history of fall from 3 meters at the age of three and she has a scar on her forehead since then. She doesn’t have any occipital trauma history. Her physical and neurological examination was normal and she didn’t have any other complaints. Her X-ray examination was evaluated as normal. On cranial CT examination we detected a bony defect of 3x3 cm (Figure 1). Her gadolinium enhanced cranial MRI revealed bony defect along with massive gadolinium enhancement in adjacent tissue (Figure 2). The lesion was located adjacent to transvers sinus, attached to dura and it was isointens in T1 weighted images.

**Surgery:** we gave the patient a prone position on operation table. We couldn’t precisely palpate the bony defect but we made a skin incision on area including the bump and dissected the cutaneous and subcutaneous tissue. We exposed the occipital bone and saw that the bony defect was 2x2 cm. We excised pathological bone tissue around the defect by use of Kerrison. The excised material was cheese-like soft. We stopped the excision at the borders of hard bone tissue. We performed a cranioplasty with Medpor at the area of defect. After the operation, we saw on cranial CT that the excision area was 4,5x4,5 cm.

**Histopathology:** on immunohistochemical examination; Material: paraffin block, Technic:Ventana-Benchmark xt, Controls: standard positive and negative, Primary antibodies(s) results have been detected as: PANCK(5/6/8/18) (scytek (5d3lp34)): negative CD45(lca) (scytek (pd7/26/162b11)): positive (mature lymphocytes) Ki-67 (dako (mib-1)): positive (%4-8: lymphocytes) S-100 (scytek (4c4.9)): positive (dendritic cells) CD68 (dako (pg/m1)): negative CD1a (novocastra (o10)): negative Vimentine (scytek (v9)): positive (diffuse; highly).

**Figure 3:**
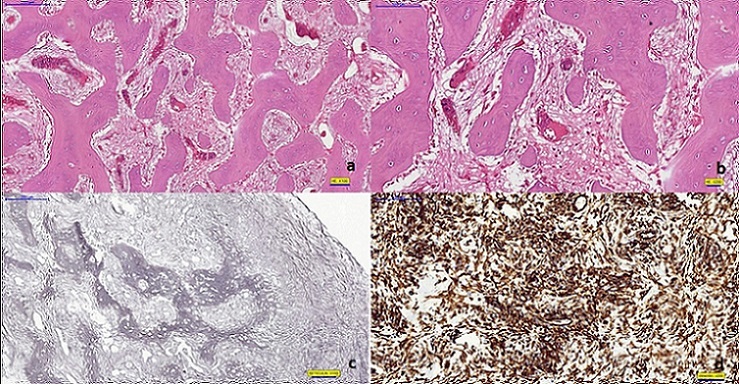
(a) fibrous dysplasia bearing “J” and “C” shaped irregular bony structures unaccompanied by osteoblastic/osteoclasticactivity. (Hematoxylin-eosin, original magnification x 100); (b)Hypercellular tissue intervening irregular shaped bony structures of fibrous dysplasia (Hematoxylin- eosin, original magnification x200); (c) Reticul in fibers forming characteristic “woven” bone pattern of fibrous dysplasia (Reticul in stain, original magnification x 100); (d) Characteristic “J” and “C” shaped irregular bony structures expressing intense and diffuse vimentin reactivity

Bone trabeculae are in shape of “c” and “j” and some of them are fused with each other (Figure 3a). Microscopically findings of the case consist of bone lesion surrounded by compact bone tissue, mature lymphocytes that infiltrate fibrous tissue and dendritic cells (Figure 3b). On polarized filter examination, it has the characteristics of “woven bone” (Figure 3c). Intertrabecular connective tissue is hypercellular. Piles of lymphocytes and dendritic cell infiltrations in fibrous tissue are remarkable. There is not any detected mitosis or necrosis. The samples of compact bone tissue are in order. Conventional histochemistry doesn’t reveal any PAS property; reticular formation with “woven” bone properties is seen on reticulin stain and collagen fibers with “woven” bone properties is seen on MTC stain (Figure 3d). With help of these findings, we diagnosed this case as a fibrous dysplasia patient.

**Follow-up:** we didn’t give the patient any adjuvant therapy. She did not have any complaints on her 3 moths follow ups. We planned to re-examine her three months later with a control cranial MRI.

## Discussion

Even though cranial fibrous dysplasia is a well-defined situation, its etiology is yet unknown. It is widely accepted that abnormal fibro-connective tissue proliferation and switch of this tissue with normal bone tissue plays role on pathogenesis [2]. Regarding to its etiology, Lichtenstein and Jaffe claimed the abnormal differentiation of mesenchyme in 1942 [6]. In 1957 Changus claimed osteoblastic hyperplasia to be the underlying pathology [7]. The frequency of regional fibrous dysplasia on cranium is controversial. At a study realized by Lustig et al. mostly affected cranial bones were reported as ethmoid (71%), sphenoid (43%), frontal (33%), maxillary (29%) and the least commons were temporal (24%) and occipital bones (5%) [8]. In another study, most commonly affected cranial bone has been stated as frontal bone and similar to the study of Lustig et al. The least commonly affected bone has been stated as occipital bone [9, 10]. The patient we report in this article is a very rare case for the localization of fibrous dysplasia. In the literature, there is a total of seven occipital fibrous dysplasia cases reported (Table 1) [11–17].

**Table 1:** Characteristics of occipital fibrous dysplasia cases in the literature

Case No	Author (year)	Age (yrs)/ Sex	Symptoms	Clinical form	Radiological type	Treatment	Additional therapy	Follow up
1	Abdelvahab et al. (1987) (11)	ND	ND	ND	ND	ND	ND	ND
2	Sato K. (1993) (12)	ND	ND	ND	ND	ND	ND	ND
3	Tajima et al. (1993) (13)	18/F	Hard, painless mass	Monostotic	Cystic	Surgery, total excision	ND	Uneventful
4	Chandy MJ. (1999) (14)	ND	Headache	Monostotic	Pagetoid	ND	ND	ND
5	Itshayek et al. (2002) (15)	19/M	Enlarging mass	Monostotic	Cystic	Surgery, biopsy	Embolization for aneurysmal bone cyst	ND
6	Liu et al. (2008) (16)	ND	ND	ND	ND	ND	ND	ND
7	Tomiyama et al. (2011) (17)	14/F	Enlarging mass	Monostotic	Cystic	Surgery, total excision	Not applicated	Uneventful
8	Presentcase	15/F	Enlarging mass	Monostotic	Cystic	Surgery, total excision	Not applicated	Uneventful

Yrs: years, ND: not described, F: female, M: male

Three forms of fibrous dysplasia have been defined by Nager. The most severe form is McCune Albright syndrome and the least severe form is monostotic type [5]. The case we present is compliant with monostotic type for showing involvement of cranial bone on one area and also for the age of diagnosis. The features of the disease depend on its localization. On craniofacial involvements, mostly, headache and atypical pain in the face is seen. On temporal bone involvements, patients complain from hearing loss due to strictures in foramen and numbness in face [8]. In occipital or parietal area involvements, clinical symptoms are usually local bumps and pain as it is in our case.

Radiologically, Fries defines fibrous dysplasia of cranium in 3 subtypes. Most common is pagetoid (56%), then sclerotic (23%) and cystic (21%) forms are seen [4]. Our case has cystic type fibrous dysplasia which is the rarest subtype. Cystic subtype is formed of many fibrous elements. Aneurysmal bone cyst, unicameral cyst, non-ossifying fibroma, Paget disease, osteocondroma, giant cell granuloma, osteosclerosis, exocytosis and osteoma are diseases to considerate for differential diagnosis. The diagnosis of fibrous dysplasia is made thanks to radiological and histopathologicinformations. The formation of woven lamellar bone and fibrous matrix is distinctive. CT is the first choice of imaging for its superiority on revealing bone tissue. CT also helps to make a differentiation between fibrous dysplasia and various bone pathologies like Paget disease or osteosclerosis [18]. MR imaging is especially used on fibrous dysplasia patients for evaluation of soft tissue, tissue adjacent to tumor and fibrous component [19]. In our case, we evaluated bone tissue, soft tissue and tissue adjacent to tumor with help of both CT and MRI.

Pathologically, fibrous dysplasia lesion is characterized by widening of cortical bone and it’s substitution with hard, rubber-like fibrous tissue [20]. Microscopically, lesion is easily detectable by irregular trabeculae of woven bone and it’s stroma of mixed connective tissue [8]. In microscopical examination of our case, we detected bone lesion in compact bone tissue and mature lymphocytes and dendritic cells showing invasion into fibrous tissue. Bone trabeculae are in shape of “c” and “j”, some of them are united with each other and there is no osteoblastic activity. On examination with polarized filter it has the characteristics of “woven bone”. Intertrabecular connective tissue is hypercellular. Except for areas of infiltration by mature lymphocytes and dendritic cells, the absence of CD1a + Langerhans cells are remarkable.

The treatment of fibrous dysplasia is a controversial subject. Only follow up is suggested for asymptomatic patients, in some cases drugs that prevent osteoblastic bone resorption are used [21]. However, in case of symptom presence, esthetical complaints and need for tissue biopsy to make a diagnose, surgery can be performed [2, 8]. Total excision of lesion results in total cure. In case of residual tissue presence or in case of non-surgical patients radiotherapy is not recommended because of malign transformation risk [9]. In our case, we performed surgery and totally resected the lesion because our patient had pain, local bump and esthetical complaint. We didn’t give the patient any adjuvant therapy.

## Conclusion

Fibrous dysplasia is a progressive bone disease of young patients. Despite its resemblance to a benign lesion by not being symptomatic it can progress and cause severe bony defects and skin lesions. Total surgical resection is necessary and sufficient for total treatment.
